# The Effect of Fatigue and Fatigue Intensity on Exercise Tolerance in Moderate COPD

**DOI:** 10.1007/s00408-016-9931-y

**Published:** 2016-08-22

**Authors:** Khaled Al-shair, Umme Kolsum, Dave Singh, Jørgen Vestbo

**Affiliations:** 1 Centre for Respiratory Medicine and Allergy, Institute of Inflammation and Repair, Manchester Academic Health Science Centre, The University of Manchester and University Hospital of South Manchester, NHS Foundation Trust, Manchester, UK; 2 Respiratory Research Group, 2nd floor The Education and Research Centre, South Manchester University Hospitals Trust, Manchester, M23 9LT UK

## Abstract

**Introduction:**

Fatigue is one of the most disabling symptoms in COPD, but little is known about the impact of fatigue on functional disability. We explored the impact of fatigue and fatigue intensity on exercise tolerance after adjusting for other factors using multivariate analysis and compared it to that of dyspnoea.

**Methods:**

A total of 119 patients with mainly moderate–severe stable COPD (38 % women, mean age 66 years) were enrolled. We used the Medical Research Council dyspnoea scores (MRC), Manchester COPD fatigue scale (MCFS) and its three dimensions, Borg scales for fatigue and dyspnoea, six-minute walk distance (6MWD), St George’s Respiratory Questionnaire, the BODE index, and the Centre for Epidemiological Study on Depression scale (CES-D), and we measured spirometry, blood gases, systemic inflammatory markers and fat-free mass index (FFMI).

**Results:**

Fatigue measured using the MCFS was associated with 6MWD and explained 22 % of the variability in 6MWD (*p* < 0.001). Fatigue remained associated with 6MWD after adjusting for MRC dyspnoea, FFMI and FEV_1_, FVC, PaO_2_, PaCO_2_, CES-D, TNF-alpha, smoking status, age and gender. We found that 33, 50 and 23 % of patients reported an increase by 2 scores on Borg scales for fatigue, dyspnoea or both at the end of the 6MWT. Fatigue scores (both before and after the 6MWT) were negatively correlated with 6MWD after adjusting for FEV_1_, FFMI, CES-D score and age (*p* = 0.007 and 0.001, respectively).

**Conclusion:**

In moderate stable COPD, fatigue may be a central driver of functional disability, to the same extent as dyspnoea.

**Electronic supplementary material:**

The online version of this article (doi:10.1007/s00408-016-9931-y) contains supplementary material, which is available to authorized users.

## Introduction

COPD is a disabling and often a progressive disease [1]. Poor exercise performance is a major manifestation of COPD, which is associated with limited functional capacity [2], impaired health status [3], depression [4–6], poor prognosis and high risk of mortality [7–9].

A limited number of studies have assessed the correlation of fatigue with functional capacity [10], and little is known about the association of fatigue with exercise intolerance using an objective measure such as the self-paced six-minute walking test (6MWT). Several other factors such as dyspnoea [5, 11], muscle mass depletion [12], age, gender [11], systemic inflammation [13, 14] and global initiative for chronic obstructive lung disease (GOLD) stages and emphysema [5] have been associated with poor performance in the 6MWT. An increase in dyspnoea is more prominent than fatigue after incremental [15, 16] and endurance shuttle walking tests [15], while fatigue is more frequently reported after cycling test [15, 17]. However, the impact of fatigue compared to dyspnoea on the performance of clinically stable COPD patients in the 6MWT has not been adequately studied.

Clinically, regular physical activities have been associated with reduced hospital admission and mortality [18], and several studies have shown that fatigue is one of the strongest factors associated with reduced physical activity and a major component of impaired health status [19–22]. Furthermore, assessment of dyspnoea severity alone may be inadequate in investigating patients suitability for pulmonary rehabilitation [23]. For these reasons, fatigue assessment may need more attention in daily clinical practice and could constitute a reasonable target for therapeutic intervention, particularly as it has been shown to improve after pulmonary rehabilitation [24, 25] and walking exercise [26].

The two main aims of this study were as follows: (1) to explore the impact of fatigue on exercise tolerance after adjusting for other factors and (2) to compare the association between fatigue intensity and walking distance in a 6MWT to that of dyspnoea intensity. We hypothesized that fatigue would impact on the 6MWD similar to dyspnoea.

## Methods

### Study Subjects

We assessed 200 patients with COPD, and 119 patients with clinically stable COPD were enrolled in the study. We excluded patients with a recent exacerbation or rescheduled their visits to ensure at least 4 weeks of clinical stability before the visit date. We also excluded patients with symptomatic ischaemic heart disease, congestive heart disease, lung cancer, known psychiatric illness and maintenance treatment with systemic corticosteroids [4]. All participants gave written informed consent to participate in the study, and the South Manchester Research Ethics Committee had approved the study (Reference number 05/Q1402/41).

### Assessments

Fatigue was assessed at enrolment using the Manchester COPD fatigue scale (MCFS) [22, 27]. It measures total fatigue as well as dimensional assessment of physical, cognitive and psychosocial fatigue. The total score ranges from 0 to 54; the higher the score, the worser the fatigue. Additionally, dyspnoea was assessed using the Medical Research Council dyspnoea scale [28]. Self-paced exercise capacity was measured using the 6MWT according to the ATS guideline [11], a cut-point for 6MWD of 350 m was chosen on the basis of the BODE index [29] and its ability to identify poor exercise performance [30]. Depressive symptoms were assessed using the Centre for Epidemiological Study on Depression (CES-D) scale [31]. We used the bioelectrical impedance analysis to measure body composition (Bodystat Ltd., Douglas, UK). Fat-free mass index (FFMI) was calculated as fat-free mass divided by height squared. For defining muscle wasting, we used the Dutch criteria: FFMI < 16 kg/m^2^ for men and FFMI < 15 kg/m^2^ for women [32]. We also assessed the intensity of fatigue and dyspnoea before and after a 6MWT using the Borg scale [33], where patients rated their perception of fatigue and dyspnoea on a scale ranging from 0 to 12, with 0 meaning no fatigue/dyspnoea and 12 meaning extreme fatigue/dyspnoea intensity [15], and it has been suggested that a change of 2 units would indicate a minimal clinically important difference in perceived dyspnoea [34, 35].

Health Status was measured using the St George’s Respiratory Questionnaire (SGRQ) [3]. The multidimensional BODE index was also used [29]. Spirometry was done according to the ATS/ERS standardisation guideline [36] using a Jaeger MasterScreen spirometer (Jaeger Ltd., Hoechberg, Germany). Earlobe capillary blood gases were measured according to the method described by Spiro and Dowdeswell [37] using a Radiometer analyzer (Radiometer Medical A/S, Copenhagen, Denmark). Pulse rate and oxygen saturation (Nonin Oximetry, Plymouth, USA) were also measured before and after 6MWT.

We also measured systemic plasma TNF-α and its receptors TNF-α R1 and R2, and serum IL-6 using high sensitivity ELISA (Quantikine, R&D Systems, Europe, Oxon, UK). Plasma CRP was measured by high sensitivity particle-enhanced immunonephelometry (Cardiophase; BN systems, Dade Behring, Newark, NJ, USA).

### Statistical Analysis

With a sample of 119 patients, the study had 80 % power to detect correlation of 0.25 or more between various variables. Normal distribution was assessed by Kolmogorov–Smirnov goodness of fit test, and non-parametric data were natural log transformed or presented as median and interquartile range (IQR). The univariate correlation of fatigue (total and dimensional MCFS scores) as well as the intensity of dyspnoea and fatigue scores (Borg scale) with the 6MWD was assessed using the Pearson and Spearman correlation, respectively. The association of walking distance (<350 vs. ≥350 m) with airflow obstruction, health status, oxygen saturation, arterial capillary blood gases, body mass and fat-free mass was examined using the independent sample *t*-tests. The Mann–Whitney test was used to investigate the association of walking distance (<350 vs. ≥350 m) with the median values of BODE, CES-D, pack-years and fatigue and dyspnoea scores in Borg scale. The difference in the median of fatigue and dyspnoea scores (Borg scale), and the mean of heart rate and O_2_ saturation before and after 6MWT were examined using the Wilcoxon test and Paired *t*-test, respectively. The independent sample *t*-test was used to examine the difference in airflow obstruction, muscle mass, health status and exercise capacity between patients who experience more fatigue, dyspnoea or both by ≥2 scores (in Borg scale) at the end of the 6MWT; similarly, the Mann–Whitney test was used to examine the difference in the median of BODE and CES-D scores. The Chi-square (χ^2^) test was used to examine the categorical association of worsening fatigue, dyspnoea or both (after 6MWT) with COPD severity (BODE quartiles), airflow obstruction (GOLD stages), exercise capacity limitation (<350 vs. ≥350 m), muscle wasting and depressive symptoms (using the 16 score cut-off for CES-D scores). Finally, multivariate linear analyses were used to examine the association of all factors with exercise capacity. SPSS version 20 (SPSS Inc, USA) was used.

## Results

We studied 119 patients with mainly moderate–severe COPD with an average age of 66 years, of which 38 % were women. As shown in Table 1, approximately half of the cohort walked less than 350 m in the 6MWT; women made up 49 % of this subgroup. These patients had worse lung function, poorer health status, more pack-years and they had relatively more dyspnoea and fatigue than those who walked ≥350 m at baseline. They also reported significantly more dyspnoea and fatigue after the 6MWT.

**Table 1:** Baseline characteristics; mean values and standard deviations are shown if nothing else is noted

	All	<350 m	≥350 m	*p* value
Number	119	59	60	–
Age, years	66 ± 6.7	65 ± 6.6	66.9 ± 6.9	0.14
Women (%)	46 (38 %)	29 (49 %)	17 (28 %)	0.03*
Ex/current smokers,	87/35	36/23	48/12	0.04*
Pack/years median (IQR)	40 (25.8)	43.6 (21)	36.6 (31.1)	0.12^#^
FEV_1_ %	52.5 ± 18.5	40.7 ± 18.8	54.1 ± 18.5	0.16
FEV_1_ (L)	1.36 ± 0.52	1.23 ± 0.52	1.5 ± 0.5	0.01
FVC (L)	3 ± 0.9	2.7 ± 0.9	3.2 ± 0.84	0.002
GOLD 1	6 (5 %)	1	5	0.07*
GOLD 2	59 (49.6 %)	26	33	0.07*
GOLD 3	38 (31.9 %)	24	13	0.07*
GOLD 4	16 (13.4 %)	7	9	0.07*
PaO_2_ (kPa)	9.2 ± 1.4	9 ± 1.2	9.2 ± 1.1	0.3
PaCO_2_ (kPa)	5.2 ± 0.58	5.3 ± 0.66	5.1 ± 0.48	0.1
sO_2_ earlobe arterial capillary blood	94 ± 2.4	93.7 ± 2.6	94.2 ± 2.1	0.3
Haemoglobin (g/dl)	14.9 ± 1.7	14.9 ± 1.7	15 ± 1.7	0.8
BMI (kg/m^2^)	27.5 ± 5.8	27.6 ± 6.1	27.4 ± 5.5	0.8
FFMI (kg/m^2^)	17.8 ± 3.1	17.7 ± 3.4	18.2 ± 3.2	0.37
SGRQ total score	46 ± 20	56.4 ± 17.4	36.6 ± 18.4	<0.001
sO_2_ before 6MWT	95.4 ± 2.8	94.9 ± 3.2	95.9 ± 2.3	0.04
sO_2_ after 6MWT	94 ± 4.5	93.7 ± 4.1	94.3 ± 4.9	0.4
Heart rate before 6MWT	74.5 ± 14	78.2 ± 12.7	70.4 ± 14.2	0.002
Heart rate after 6MWT	89.9 ± 17.2	91.2 ± 13.5	88.7 ± 19.3	0.39
Dyspnoea (Borg Scale) before 6MWT (median (IQR)	0.5 (1)	0.5 (2.1)	0.3 (0.75)	0.02^#^
Dyspnoea (Borg Scale) after 6MWT (median (IQR)	3 (3)	3 (2)	2 (2.5)	0.003^#^
Fatigue (Borg Scale) before 6MWT (median (IQR)	0.5 (2)	0.75 (2.5)	0.1 (1)	0.001^#^
Fatigue (Borg Scale) after 6MWT (median (IQR)	2 (3.5)	3 (2.25)	0.7 (2)	<0.001^#^

*IQR* interquartile range, *FEV*_*1*_ *%* forced expiratory volume in 1 s in % of predicted, *FVC* forced vital capacity, *PaO*_*2*_ arterial oxygen partial pressure, *PaCO*_*2*_ arterial carbon dioxide partial pressure, *BMI* body mass index, *FFMI* fat-free mass index, *GOLD* global initiative for chronic obstructive lung disease, *SGRQ* St George’s Respiratory Questionnaire

^*^ χ^2^-test

^#^ Mann–Whitney *U* test

Exercise tolerance was negatively correlated with physical, cognitive and psychosocial fatigue dimensions of the MCFS (*r* = −0.46, −0.40 and −43, respectively; *p* < 0.0001 for all three measures). Total MCFS scores had the highest negative correlation with exercise capacity (*r* = −0.47, *p* < 0.0001), and it explained 22 % of the variance of exercise tolerance. We found that TNF-α only had a negative correlation with 6MWD (*ρ* = −0.2, *p* = 0.047), and CRP trended to correlate with 6MWD (*ρ* = −0.18, *p* = 0.065). In a fully adjusted model including MRC dyspnoea scale, FFMI, FVC, sO_2_, CES-D and FEV_1_ %, the model explained 38 %; this model was the best at explaining the variation in exercise tolerance (see Table 1 in the e-supplement).

Univariate analysis showed that fatigue and dyspnoea scores measured with the Borg scale had similar negative correlations with 6MWD, before (*ρ* = −0.35 and −0.26; *p* = <0.0001 and 0.006, respectively) as well as after the 6MWT (*ρ* = −0.4 and −0.031; *p* = <0.0001 and <0.001, respectively). Both dyspnoea and fatigue intensity scores were significantly higher after the test as shown in Table 2.

**Table 2:** Dyspnoea, fatigue, heart rate and O_2_ saturation before and after the 6MWT

	Before	After	*p* value
Dyspnoea (Borg Scale) [median (IQR)]	0.5 (0–1.1)	3 (1–4)	<0.001
Fatigue (Borg Scale) [median (IQR)]	0.5 (0–2)	2 (0.5–4)	<0.001
Heart rate [mean (SD)]	74.5 (14)	90.1 (16.6)	<0.001
Oxygen saturation [mean (SD)]	95.4 (2.8)	94 (4.5)	<0.001

**Fig. 1:**
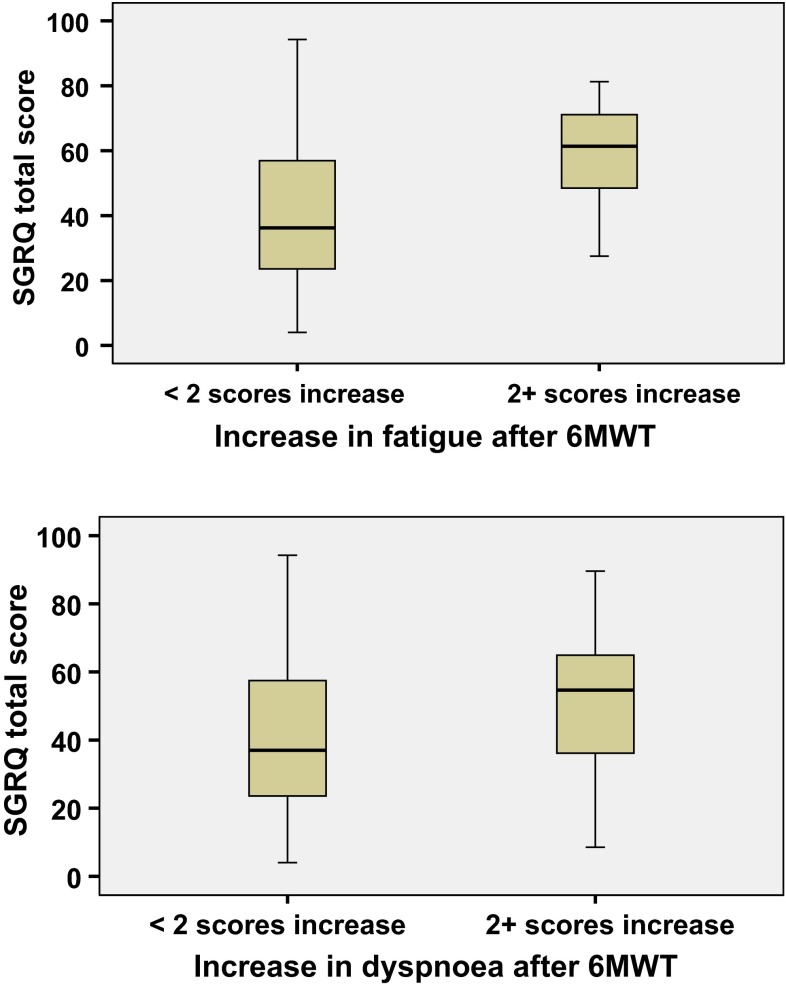
The association of worsening in fatigue and dyspnoea (after 6MWT) and health status

We subsequently categorized patients according to whether or not they reported an increase ≥2 scores on the Borg scale after the 6MWT (more fatigued and more dyspnoeic). We found that 33, 50 and 23 % of our samples reported an increase by 2 or more scores for fatigue, dyspnoea or both after the 6MWT. These patients had comparably poorer 6MWD, more severe COPD (FEV_1_ % or BODE scores), more depression and worse quality of life, as illustrated in Fig. 1 and Table 3.

**Table 3:** The relationship of worsening in fatigue and dyspnoea or both (post-6MWT) to COPD measurements

	Worsening fatigue by <2 scores	Worsening fatigue by ≥2 scores [*n* = 37, (32 %)]	*p*	Worsening dyspnoea by <2 scores	Worsening dyspnoea by ≥2 scores [*n* = 57 (49 %)]	*p*	Worsening dyspnoea and fatigue by <2 scores	Worsening dyspnoea and fatigue by ≥2 scores [*n* = 27 (23 %)]	*p*
BODE [median (IQR)]	2 (1–3)	4 (2–5)	<0.001	2 (1–3)	3 (1–5)	0.04	2 (1–4)	4 (2–5)	0.013
FEV_1_ % [mean (SD)]	54.7 (17.7)	46.7 (19.4)	0.03	55.5 (17.3)	48.5 (19.2)	0.04	53.2 (17.7)	48.6 (21)	0.3
Muscle mass (FFMI)	17.8 (3.2)	18.1 (3.7)	0.64	17.5 (3)	18.5 (3.7)	0.12	17.9 (3.2)	18.3 (3.9)	0.6
Total SGRQ [mean (SD)]	40 (20.6)	59 (15)	<0.001	40.0 (20.5)	52.2 (19.7)	0.004	42.6 (20.6)	59.8 (15.4)	<0.001
CES-D [median (IQR)]	8 (3–14)	13 (9–18)	0.004	7.5 (2–13.5)	12 (6–18)	0.004	8 (2–14)	14 (11–20)	<0.001
6MWD [mean (SD)]	361.7 (69)	316 (72.4)	0.002	354.5 (70.3)	340.7 (75)	0.3	354.5 (71)	324.1 (15.6)	0.07

BODE: multidimensional index (*B* body mass index, *O* obstruction of airways as measured by FEV_1_, *D* dyspnoea as measured by MRC scale, *E* exercise capacity as measured by 6MWT); *CES-D* Centre for Epidemiologic Studies Depression Scale, *FEV*_*1*_ *%* forced expiratory volume over 1 s of predicted, *FFMI* fat-free mass index, *6MWD* 6 Minute walk distance, *m* metre, *IQR* interquartile range, *SGRQ* George’s Respiratory Questionnaire

Moreover, categorical analysis showed that patients who experienced more fatigue and dyspnoea intensity after 6MWT had more severe illness, more depression or more impaired self-paced exercise performance as shown in Table 4.

**Table 4:** The relationship of worsening in fatigue and dyspnoea or both (post-6MWT) with COPD measurement

	Worsening fatigue by ≥2 scores [*n* = 37, (32 %)]		Worsening dyspnoea by ≥2 [*n* = 57 (49 %)]		Worsening dyspnoea and fatigue by ≥2 scores [*n* = 27 (23 %)]	
	OR (95 % CI)	*p*	OR (95 % CI)	*p*	OR (95 % CI)	*p*
BODE quartiles (Q1,2 vs. 3,4)	6.9 (2.5–19.1)	<0.001	3.6 (1.3–10)	0.02	4.9 (1.8–13.2)	0.003
GOLD stages (1,2 vs. 3,4)	2.5 (1.14–5.63)	0.04	1.9 (0.9–4)	0.12	1.5 (0.6–3.5)	0.5
Depressed versus not depressed	2.6 (1.1–6.2)	0.05	2.5 (1–6)	0.06	3.4 (1.36–8.7)	0.02
6MWD <350 versus ≥350 m	0.35 (0.16–0.8)	0.02	0.66 (0.3–1.4)	0.35	0.5 (0.21–1.22)	0.2

BODE: multidimensional index (*B* body mass index, *O* obstruction of airways as measured by FEV_1_, *D* dyspnoea as measured by MRC scale, *E* exercise capacity as measured by 6MWT), *GOLD* global initiative for chronic obstructive lung disease, *6MWD* 6 minute walk distance, *m* metre

Using 6MWD as a dependent variable, multivariate models of fatigue and dyspnoea before the 6MWT showed little difference, *β* = −0.20 (*p* = 0.06) for fatigue and −0.21 (*p* = 0.05) for dyspnoea; however, a stronger association for fatigue than for dyspnoea was seen after the 6MWT, *β* = −0.32 (*p* = 0.004) and −0.15 (*p* = 0.17), respectively. Moreover, fatigue scores remained associated with 6MWD after adjusting for FEV_1_ %, FFMI, CES-D score and age, *β* = −0.25 (*p* = 0.007) before the 6MWT and −0.30 (*p* = 0.001) after.

## Discussion

We have reported two novel findings. First, fatigue had a significant burden on self-paced exercise capacity and contributed significantly to explaining the variance of limited exercise capacity. These findings were consistently seen in univariate analysis and in multivariate analysis modules after adjusted for several confounding factors. Second, using univariate and multivariate analyses, the impact of fatigue intensity on exercise performance (before and after the 6MWT) was at least comparable to the impact of dyspnoea intensity.

Fatigue impact seems a central driver of disability as it alone explained 22 % of the variance of limited exercise capacity. This association remained statistically significant after adjusting for several confounding factors such as dyspnoea [11], muscle mass depletion [12], depressive symptoms [4], lung function deterioration, age, gender [11] and markers of systemic inflammation [13, 14], where TNF-α only remained statistically significant in multivariate analyses (Table S1). The best multivariate module explained 38 % of limited exercise performance. In fact, our finding suggests that there are factors, other than fatigue and the variables we have included, that affect exercise performance and functional capacity such as muscle weakness [38], left cardiac dysfunction [39] and degree of emphysema [5]. Univariate and multivariate analyses showed that fatigue had at least comparable impact on the self-paced exercise capacity as dyspnoea. Fatigue is a complex phenomenon, and its impact on exercise capacity in COPD can be due to the poor quality of sleep [40], metabolic skeletal muscle stress [41], lack of energy and depressive symptoms [27], the impact of the labour of breathing [42], linkage with exacerbation frequency [43] and severity [44], lack of regular physical activity [25], muscle depletion and dysfunction [45], and possibly systemic inflammation [46].

The choice of exercise test is important for understanding the results as dyspnoea has previously been shown to be more prominent after the ISWT [15, 16] and the ESWT [15], whereas fatigue was more prominent after a cycling test [15, 17]. The 6MWT is a robust and simple test that is widely used with the advantage that patients familiarly walk at their own pace [11]. Therefore, the test likely reflects the daily functional capacity [2] and the whole body functional performance [11]. Additionally, the test has been widely validated, and a poor performance in the test has frequently shown a significant association with deteriorated quality of life [11], low mood [4], depleted muscle mass [12], poor prognosis [30] and mortality [8].

Our findings suggest that an improvement in fatigue after medical intervention such as pulmonary rehabilitation would result in a significant improvement in quality of life. Indeed, fatigue seems to improve after pulmonary rehabilitation [24, 25]. However, the scales used in assessing the response of fatigue to pulmonary rehabilitation are general scales, and fatigue is a complex phenomenon that needs further investigation in larger studies with disease-specific robust scales, e.g., the Manchester COPD fatigue scale [27]. Second, the finding of this study supports the idea that dyspnoea severity alone (usually grade 3–5) is likely inadequate to assess the suitability of patients for pulmonary rehabilitation [47]. Indeed, patients would achieve a considerable benefit from pulmonary rehabilitation regardless of their MRC dyspnoea grade [23]. Therefore, other factors such as fatigue, depression and possibly muscle wasting should be considered in assessing the suitability of patients for pulmonary rehabilitation, particularly since these factors often affect the motivation of the patients to complete pulmonary rehabilitation program [48].

In conclusion, using two validated tools in measuring fatigue impact and intensity in COPD, this study quantified the substantial effect of fatigue on self-paced exercise capacity. In comparison to a similar dyspnoea worsening, fatigue worsening after 6MWT was significantly associated with poor exercise performance in moderate–severe stable COPD. This finding highlights a need for assessing fatigue in more depth in future COPD studies.

## Electronic Supplementary Material

Below is the link to the electronic supplementary material.

Supplementary material 1 (DOCX 15 kb)
