# Supplementary material for: The Effect of Fatigue and Fatigue Intensity on Exercise Tolerance in Moderate COPD
Source: Lung. 2016 Aug 22;194(6):889–95. doi: 10.1007/s00408-016-9931-y (PMC5093188; doi:10.1007/s00408-016-9931-y)
Supplement: Supplementary file 1 — Supplementary material 1 (DOCX 15 kb) [file 408_2016_9931_MOESM1_ESM.docx]

**The effect of fatigue and fatigue intensity on exercise tolerance in moderate COPD**

Khaled Al-shair, Umme Kolsum, Dave Singh, Jørgen Vestbo

Results:

Table 1, Multivariate linear regression modules for factors associated with exercise intolerance

| Module 1 | | | | Module 2 | | | | Module 3 | | | | **Final Module** | | | |
| --- | --- | --- | --- | --- | --- | --- | --- | --- | --- | --- | --- | --- | --- | --- | --- |
| Variables | Beta | p | R^2^ | Variables | Beta | p | R^2^ | Variables | Beta | p | R^2^ | **Variables** | **Beta** | **p** | **R^2^** |
| FEV_1_% | 0.008 | 0.9 | 18% | Age | 0.06 | 0.51 | 37% | MRC dyspnea | -0.26 | 0.009 | 37% | **MRC dyspnea** | **-0.27** | **0.006** | **38%** |
| FVC | 0.3 | 0.002 |  | Gender | -0.09 | 0.4 |  | MCFS | -0.23 | 0.034 |  | **MCFS** | **-0.2** | **0.048** |  |
| PaO_2_ | -0.1 | 0.5 |  | CES-D | -0.2 | 0.2 |  | TNF-α | -0.18 | 0.037 |  | **FVC** | **0.18** | **0.053** |  |
| PaCO_2_ | -0.08 | 0.4 |  | MCFS | -0.23 | 0.03 |  | FVC | 0.19 | 0.038 |  | **sO_2_** | **0.22** | **0.009** |  |
| sO_2_ | 0.33 | 0.02 |  | MRC dyspnoea | -0.27 | 0.004 |  | sO2 | 0.15 | 0.09 |  | **FFMI** | **0.1** | **0.24** |  |
|  |  |  |  | FFMI | 0.11 | 0.2 |  | FFMI | 0.13 | 0.12 |  | **CES-D** | **-0.13** | **0.2** |  |
|  |  |  |  | Smoking status | -0.07 | 0.4 |  | CES-D | -0.01 | 0.9 |  | **FEV_1_%** | **-0.03** | **0.7** |  |
|  |  |  |  | Pack/years | -0.12 | 0.18 |  | FEV_1_% | -0.014 | 0.87 |  |  |  |  |  |
